# Moderate maternal nutrient reduction in pregnancy alters fatty acid oxidation and RNA splicing in the nonhuman primate fetal liver

**DOI:** 10.1017/S204017442300003X

**Published:** 2023-03-16

**Authors:** Kip D. Zimmerman, Jeannie Chan, Jeremy P. Glenn, Shifra Birnbaum, Cun Li, Peter W. Nathanielsz, Michael Olivier, Laura A. Cox

**Affiliations:** 1 Center for Precision Medicine, Wake Forest University School of Medicine, Winston-Salem, NC, USA; 2 Southwest National Primate Research Center, Texas Biomedical Research Institute, San Antonio, TX, USA and; 3 Animal Science, University of Wyoming, Laramie, WY, USA

**Keywords:** Developmental programming, RNA-seq, fatty acid beta-oxidation, RNA splicing

## Abstract

Fetal liver tissue collected from a nonhuman primate (NHP) baboon model of maternal nutrient reduction (MNR) at four gestational time points (90, 120, 140, and 165 days gestation [dG], term in the baboon is ~185 dG) was used to quantify MNR effects on the fetal liver transcriptome. 28 transcripts demonstrated different expression patterns between MNR and control livers during the second half of gestation, a developmental period when the fetus undergoes rapid weight gain and fat accumulation. Differentially expressed transcripts were enriched for fatty acid oxidation and RNA splicing-related pathways. Increased RNA splicing activity in MNR was reflected in greater abundances of transcript splice variant isoforms in the MNR group. It can be hypothesized that the increase in splice variants is deployed in an effort to adapt to the poor *in utero* environment and ensure near-normal development and energy metabolism. This study is the first to study developmental programming across four critical gestational stages during primate fetal liver development and reveals a potentially novel cellular response mechanism mediating fetal programming in response to MNR.

## Introduction

The fetal origins of health and disease hypothesis suggests that poor maternal nutrition during pregnancy can alter the trajectory of fetal development and adversely impact later-life health.^1^ However, most cases involve the more subtle effects of moderate fetal nutrient reduction that often remain latent until adulthood.^2^ A maternal diet lacking appropriate amounts of nutrients and calories is a major contributor to adverse pregnancy outcomes and the development of intrauterine growth restriction (IUGR). Due to the challenge of studying the molecular mechanisms underlying the development of IUGR and other fetal changes in pregnant women, we have developed a well-characterized baboon nonhuman primate (NHP) model of maternal nutrient reduction (MNR) to determine the molecular changes involved in fetal tissues including the developing fetal liver.^3^ We hypothesize that there are significant and potentially long-lasting changes to the cellular machinery (e.g., transcriptional and epigenetic mechanisms) that are induced *in utero* by poor maternal nutrition.

In this study, we examined liver transcriptional changes across four different gestational time points, covering the second half of primate fetal development. The liver is an important organ to study because the liver plays two critical roles in the fetus as a vascular connection between the developing placental vessels to the heart and the location where blood stem cells reside prior to the development of the bone marrow. At birth, the liver is responsible for cholesterol synthesis and transport, glycogen synthesis and storage, detoxification, and metabolism. Our untargeted characterization of transcriptional changes in MNR of fetal livers strongly suggests that the nutrient restriction (NR) impacts energy and nutrient utilization by altering the expression of genes involved in fatty acid beta-oxidation. Our results also suggest the fetal liver is utilizing a novel mechanism to adapt to the nutrient-poor environment by increasing abundance of alternative splice isoforms.

## Method

### Animal care and maintenance

All procedures were approved by the Texas Biomedical Research Institute (TBRI) Institutional Animal Care and Use Committee and conducted in facilities approved by the Association for Assessment and Accreditation of Laboratory Animal Care. The study was carried out in compliance with the ARRIVE guidelines. Procedures were performed by a Southwest National Primate Research Center veterinarian at TBRI. Baboons were housed in social groups to permit normal physical and social interaction. Details of housing structure and environmental enrichment provided have been published elsewhere.^4^ Social group structure, pregnancy timing, feeding system adaptation, and food intake monitoring with the individual feeding system have been described previously.^4^ Standard monkey chow biscuits were provided (Purina Monkey Diet 5038). To ensure homogeneity of females assigned to either the control group or nutrient reduction group we considered a number of morphometric measurements made prior to pregnancy, including weight, length, and BMI. Nonpregnant female baboons were fed *ad libitum* until 30 dG when they were assigned at random to control (CON) or MNR groups. Control dams continued to feed *ad libitum* and MNR dams were fed 70% of feed consumed by controls at the same stage of gestation on a weight adjusted basis. Water was continuously available in individual feeding cages and with lixits at multiple locations in the group housing.

**Fig. 1.**
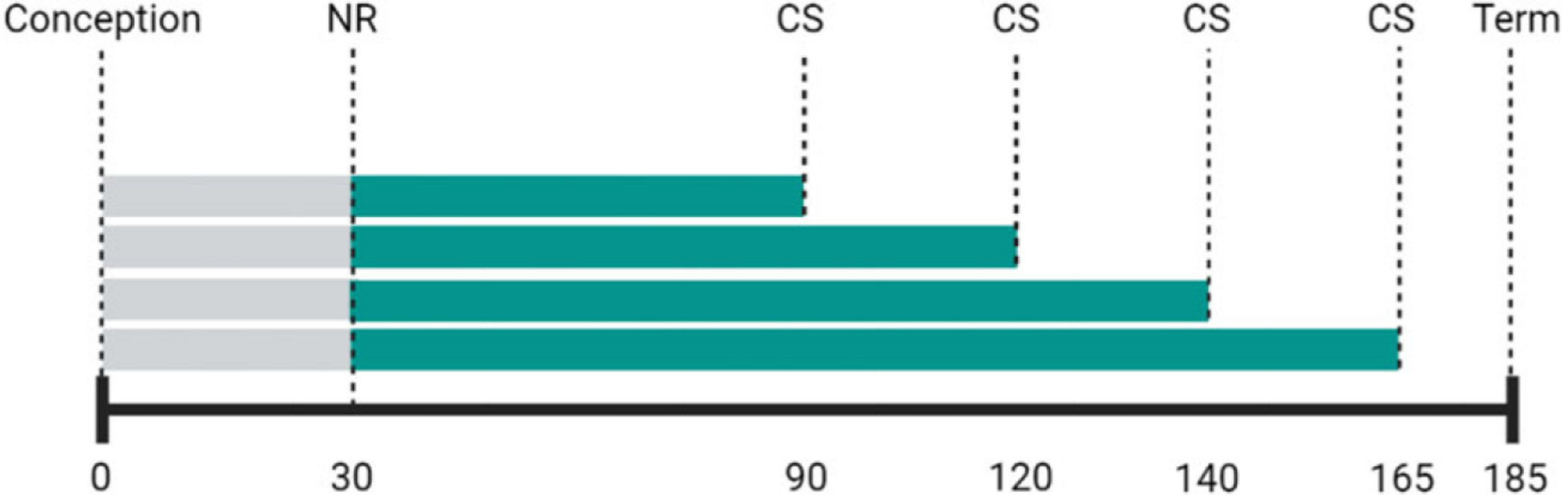
Experimental design. Fetal Liver Tissue was collected by cesarean section (CS) at 90 days gestation (dG), 120, 140, and 165 dG. Maternal nutrient restriction (NR) was introduced at 30 dG. Full term is 185 days.

Caesarean sections (CSs) were performed under isoflurane anesthesia (2%, 2 l × min^−1^) to obtain the fetus and placenta at 90, 120, 140, and 165 dG (Fig. 1).^5^ Techniques used and postoperative maintenance have been previously described in detail.^4^ Analgesia was provided with buprenorphine hydrochloride at 0.015 mg kg^−1^ day^−1^ during three postoperative days (Buprenex^®^ Injectable, Reckitt Benckiser Health care (UK) Ltd, Hull, UK). At each of the 90 and 165 dG time points, eight control samples and eight MNR samples were collected. At each of the 120 and 140 dG time points, six control samples and six MNR samples were collected. Each group had an equal number of males and females. Fetal morphometrics were obtained at the time of CS.

### RNA isolation

Fetal livers were collected at necropsy and the three lobes separated. One half of each lobe was immediately snap frozen in liquid nitrogen and then stored at −80°C until used for RNA extractions. Total RNA was isolated from each tissue using TRIzol Reagent (Invitrogen, Carlsbad, CA) as described (PMID: 23637735). RNA quantity and quality were assessed spectrophotometrically using a NanoDrop^™^ 8000 (Thermo Fisher Scientific, Wilmington, DE). RNA integrity was also confirmed by electrophoresis in a denaturing agarose gel, and extracts were stored at −80°C until use.

### Sequencing

RNA samples were used to generate cDNA libraries using Illumina’s mRNA-Seq Sample Preparation Kit according to the manufacturer’s protocol. In brief, mRNA was purified from each RNA sample using poly-A selection, chemically fragmented into small pieces, and copied into first strand cDNA using random hexamer priming. Second strand cDNA synthesis was carried out using DNA Polymerase I and RNase H. Each cDNA library was then hybridized to an individual lane of a flow cell for cluster generation using the Illumina Paired-End Cluster Generation Kit v4 and Cluster Station and subsequently sequenced using the Illumina v4 Sequencing Kit and GAIIx Sequencer using a 101-cycle paired-end sequencing run.

### Data analysis

After removing low quality bases (Phred score <30) from fastq files, trimmed reads were aligned using STAR^6^ to the olive baboon reference (Panu_3.0, GCF_000264685.3). Aligned reads were quantified using an expectation-maximization algorithm^7^ with the Panu_3.0 annotation file (release 103 from NCBI) in Partek Flow (St. Louis, Missouri). Only paired-end reads with 100% of read length overlapping with transcripts were counted. Junction reads were counted if the skipped sequences matched introns of the transcripts.

Raw read counts were filtered to remove transcripts that had a maximum read count across all samples <15. This was done to remove lowly expressed genes in order to stabilize the estimation of the mean–variance relationship with variance modeling at the observational level (“voom”) (Fig. S1).^8^ Differential expression analysis was computed with limma-voom which allows for more complex models than other differential expression analysis tools. A single model was fit on the normalized read counts. Gene expression was modeled as the outcome and sex, time, time-squared, and MNR status were modeled as predictors. The interactions between time and MNR status and time-squared and MNR status were also included in the full model. Contrasts for the MNR term as well as the two interaction terms were computed for each gene and output separately. For each of the three primary contrasts of interest a Benjamini–Hochberg false discovery rate (FDR) was applied to the unadjusted *p*-value to account for multiple comparisons.^9^ After correction for multiple comparisons, transcripts from the same gene symbol were removed, and only the most significant transcript was retained to reduce redundancy in the pathway analysis. Transcripts meeting an unadjusted p-value <0.001 (FDR <0.15) were retained for downstream pathway analysis with transcripts meeting an FDR <0.1 being prioritized for biological interpretation (Table S1).

The gene symbol for each transcript was provided to STRING to obtain all of the known protein–protein interactions among the list of significantly differentially expressed genes.^10^ Default settings were used when searching for protein–protein interactions. MCODE was applied to the results to identify densely connected regions among the network of protein–protein interactions, and Cytoscape was used to visualize the resulting networks.^11^ Gene Ontology was used to identify the biological function of each MCODE cluster that had an MCODE score >4, contained more than six nodes, and included at least one gene that had an FDR adjusted p-value <0.1.^12,13^

Since some of the significantly differentially expressed genes were identified in connection to “RNA splicing” pathways, we summed up the number of unique transcripts for each gene in each sample to discover if any genes were being spliced differently between conditions. For each individual time point, Poisson regression was then computed on the number of unique transcripts for each gene, where transcript number was modeled as the outcome and MNR status was modeled as predictor, adjusting for sex as a covariate. Because the majority of the differentially expressed genes from the quadratic analysis demonstrated the largest differences at 120 and 140 dG, an inverse variance-based meta-analysis was computed for each gene across 120 and 140 dG.^14^ The resulting p-values were adjusted with a Benjamini–Hochberg FDR to account for multiple comparisons.^9^

## Results

**Fig. 2.**
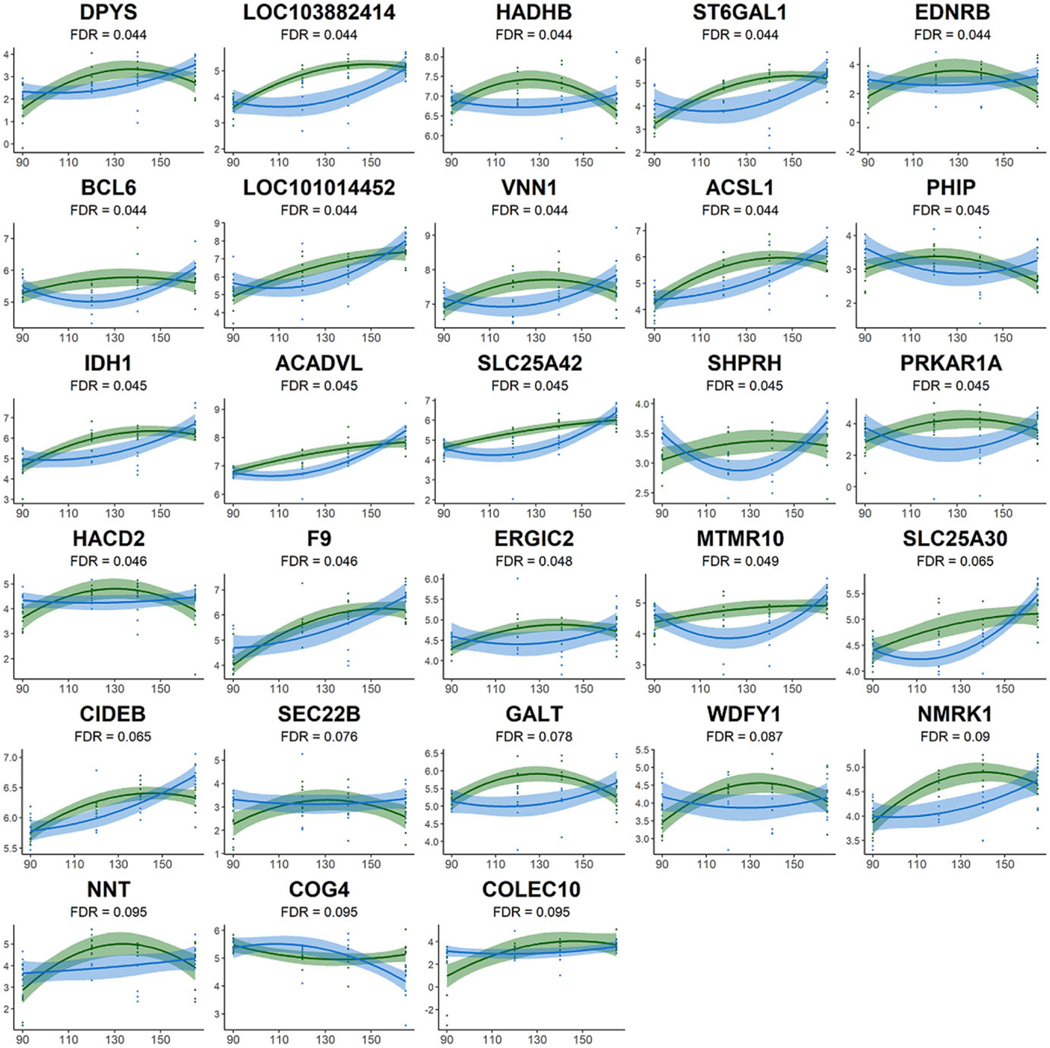
Differentially expressed transcripts (FDR < 0.1) for the quadratic interaction between time-squared and MNR status. The change in expression over time is visualized for each transcript, which is listed with the gene symbol above each figure. Controls are colored green, while MNR animals are colored blue. Transcripts are sorted by significance with the most significant transcripts at the top left.

70,853 transcripts were identified with at least one read across all baboon liver samples studied. Transcripts that did not have a maximum read count more than 15 across all samples were removed to ensure an accurate modeling of the mean–variance relationship (Fig. S1). After filtering, 14,078 transcripts remained for hypothesis testing. Post hoc, less significant transcripts with duplicated gene symbols were also removed leaving data on 8754 expressed genes. 12 genes met an FDR <0.1 for the MNR contrast (Table S1), no genes met an FDR <0.1 for the linear interaction between MNR status and time, and 28 genes met an FDR <0.1 for the quadratic interaction between MNR status and time-squared (Table S1). The majority of significant changes were from the contrast of the quadratic interaction term, and nearly all of the genes identified by the MNR contrast were also identified by the quadratic interaction (Fig. 2; Table S1). This indicates that instead of expression differences continuing to increase over time between the two groups, the majority of transcripts deviate most at the middle time points before returning to similar relative expression values between groups (Fig. 2). This is important because it suggests that 120 and 140 dG may be critical developmental windows where the expression of these genes differs markedly in the MNR fetal livers before their relative expression matches the expression level in the controls at 165 dG.

In order to complete network analysis we included an additional 56 genes that met an unadjusted p-value <0.001 (FDR <0.15) along with the 28 genes already meeting an FDR <0.1 (Fig. 2). This was done to provide additional supporting evidence to the handful of genes meeting the more stringent FDR criterion. We recognize that by expanding this gene list we are introducing potential false positives, but since false positives are expected to be random, we anticipate that most of the false positives not cluster during pathway analysis. Because of the high amount of overlap between the results of the two contrasts with significant genes, pathway analysis revealed identical networks whether or not the genes from the MNR contrast were included in the analysis.

**Fig. 3.**
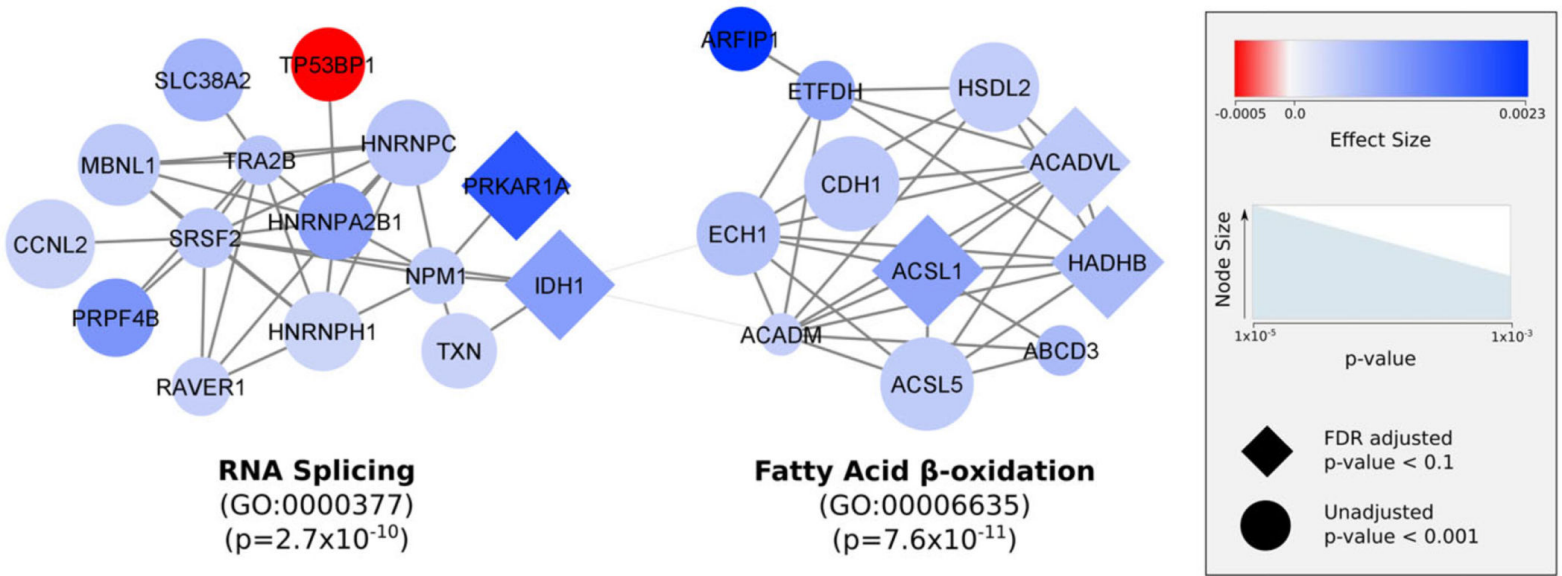
Protein–protein interaction network of differentially expressed genes significantly (*p*< 0.001) altered between MNR and control animals over time. Protein interactions were obtained from STRING’s protein interaction database. MCODE was used to find tightly connected clusters of interactions that are labeled according to function defined in gene ontology biological processes. Differences in expression values are visualized by the blue-to-red color scale. Red indicates a positive effect size, which means that expression initially decreases during the 90–120 dG window and then increases during the 140–165 dG window for the MNR group while the control group shows the opposite pattern. Blue indicates the opposite pattern between the MNR and control group. The size of the node reflects statistical significance, and diamonds represent genes that meet an FDR-adjusted *p*-value <0.05.

Network analysis revealed two key networks that had MCODE scores >4, consisted of more than six nodes, and included at least one gene that met an FDR <0.1. One network was significantly enriched for the fatty acid beta-oxidation pathway, and the other network consisted of genes involved in the regulation of RNA splicing (Fig. 3). The genes in these pathways were all downregulated in the MNR animals during the 120 and 140 dG window, with TP53BP1 being the only exception where expression was upregulated (Fig. 3). These results suggest two main pathways that are significantly affected by MNR in the fetal liver during development.

**Fig. 4.**
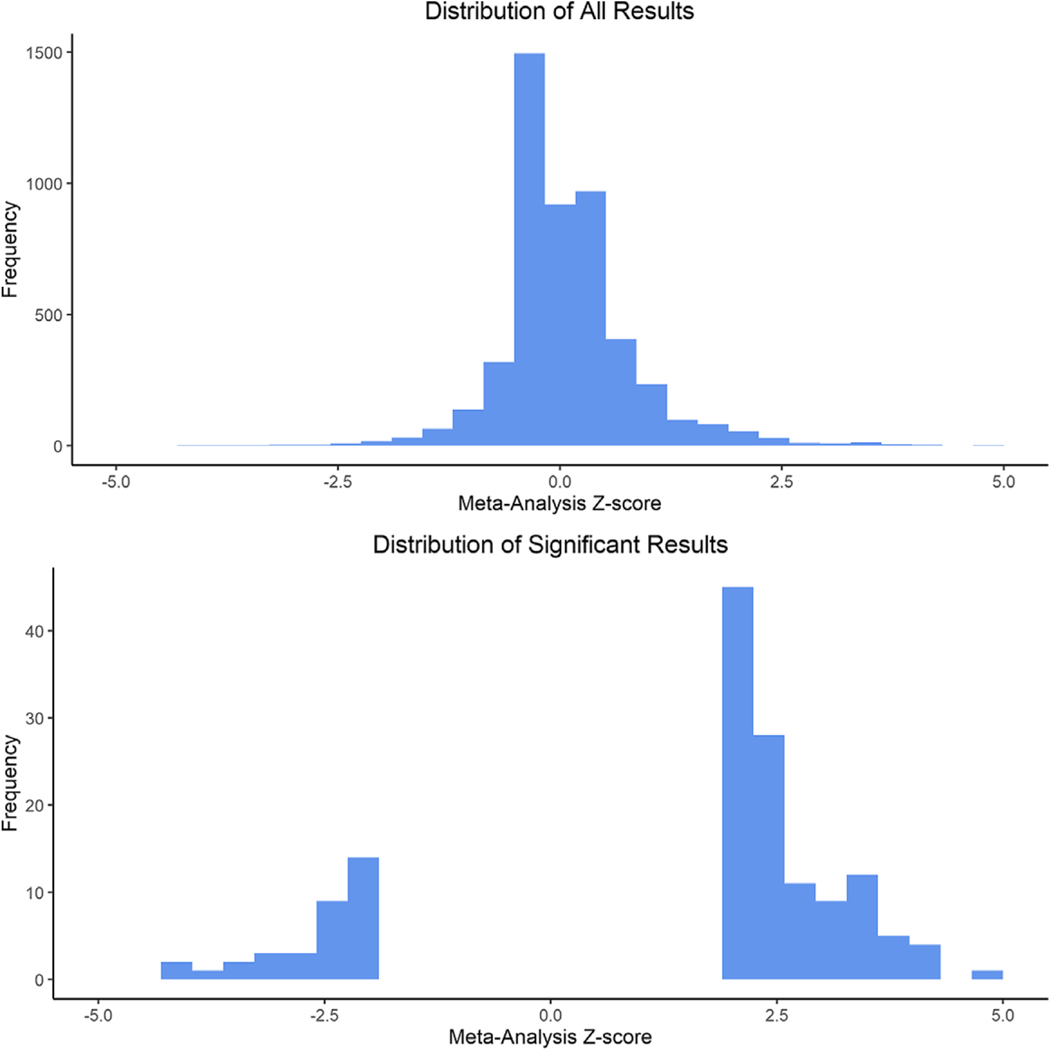
Skewed distribution of differentially spliced genes altered between MNR and control animals at 120 and 140 dG. the top histogram displays all meta-analysis Z-scores from differential splicing analysis, while the bottom histogram displays only results that meet a *p*-value < 0.05. The meta-analysis Z-score combines effect sizes from differential splicing analysis done at 120 and 140 dG. The distribution of Z-scores is skewed in favor of positive Z-scores (mean = 0.078, skewness = 1.01, *t*-test *p*-value that mean is not equal to zero = 4.7 × 10^−13^) indicating that more results demonstrate a positive increase in splice variants in MNR samples.

Given one of the networks identified by our analysis involved potential changes to RNA splicing and processing machinery, we attempted to validate this finding by examining if there is a change in the numbers of splice isoforms expressed for all or a subset of genes. Therefore, we tested for differences in the abundances of splice variants between the MNR and control groups. For each gene, we counted the number of unique transcripts in each individual sample. Poisson regression, adjusting for sex as a covariate, was used to identify genes with significantly different numbers of unique transcripts per gene. Each of the four time points was analyzed independently to identify genes that had significantly altered numbers of splice variants between the two groups. A meta-analysis was computed between the results from the 120 and 140 dG analyses since those were the two time points demonstrating the most drastic differences in expression of the splicing-related genes. At 90 dG we identified nine genes (CADPS, CUNH22orf15, RALGPS1, LOC101004909, ABCC8, RBFOX3, SNAP91, LOC103886823, TNNT3) with differential numbers of unique transcripts between groups (FDR <0.05) (Fig. S2). Five of the genes showed more splice variants in the MNR group, while four showed more in the control group. At 120 dG we identified five differentially spliced genes (MYT1L, ENOX1, LOC103886823, EBF3, LRRC7), and all of them increased in the MNR group (Fig. S3). Again, at 140 dG we identified five differentially spliced genes (ABLIM1, PTPRT, ENOX1, LY6H, LOC103880245) and all of them increased in the MNR group (Fig. S4). At 165 dG, only PTPRT was identified as significantly differentially spliced between groups (Fig. S5). For the meta-analysis across both 120 and 140 dG, we identified four genes (ENOX1, LY6H, EBF3, and ABLIM2) with differential numbers of unique transcripts and all of them showed more splice variants in the MNR group. The meta-analysis also demonstrated a general increase in the number of splice variants in the MNR groups for a majority of genes (p = 4.7 × 10^−13^) (Fig. 4). The shift is more apparent for those results that meet an unadjusted p-value <0.05 (Fig. 4, lower panel).

In all, we discovered several genes that are differentially expressed during the development of the fetal liver between MNR and control. These genes all demonstrate an interesting pattern where, compared to controls, their relative abundance decreases in the MNR animals at 120 and 140 dG but returns to similar amounts pre-term (165 dG). These genes are involved in two main pathways – fatty acid beta-oxidation and the regulation of RNA splicing. In addition, the downregulated expression patterns of the splicing-related genes are reflected in the globally increased amounts of RNA splice variants present in the MNR fetal liver transcripts at 120 and 140 dG compared to the control group.

## Discussion

Differences in the metabolism and fetal development were, for a long time, primarily attributed to genetics. However, compelling evidence shows that the intrauterine environment and maternal diet play critical roles in fetal development, with potential effects on the developing individual throughout life.^2^ Fetal programming has been a topic of increasing interest in the last decade, and the explosion of omics technologies have enabled deep analyses of the potential underlying molecular mechanisms.^15,16,17^ Given the challenges of studying fetal programming in pregnant women, we have developed a well-established NHP model of MNR in which we have evaluated programming effects on the endocrine,^15^ cardiovascular,^5^ neurological,^18^ and hepatic metabolic systems.^3^ Here we utilize this model to directly analyze the effects of MNR on the developing fetal liver transcriptome. This is the first study to characterize developmental programming of the transcriptome across multiple gestational time points in the developing primate liver. By unraveling how changes to the intrauterine environment specifically alter transcriptional machinery in the liver, we provide further insight into how maternal nutrition can directly affect the developing fetus and speculate on potential long-term health consequences as a result of these early functional changes in a key organ regulating carbohydrate and lipid metabolism.

Our current study demonstrates that moderate MNR causes specific transcriptional alterations with much larger impact at 120 dG and 140 dG than 90 dG or 165 dG. We identified no significant MNR- or control-related linear associations between transcript abundance over the developmental period studied. Instead, the quadratic interaction between MNR status and time-squared unveiled 28 genes with transcripts that differed significantly (FDR <0.1) in their expression patterns during the observed gestational period (Fig. 2). The correlation of transcriptional changes with time-squared likely indicates that fetal liver transcription in both controls and MNR fetal livers is tightly regulated throughout gestation, and transcription only changes between MNR and controls during very specific time windows, not continuously throughout gestation. These time windows may be critical in establishing energy metabolism and storage during fetal development for postnatal survival. The majority of the quadratic interaction results had positive effect sizes, indicating that in the response to the challenge of MNR, expression initially decreases during the 90–120 dG window and then increases during the 140–165 dG window for the MNR group while the control group shows the opposite pattern (Fig. 2). These significant differences in expression trajectories over time reflect key transcriptional differences in the fetal liver during a period of rapid weight gain and critical energy storage.

The majority of these results show 120 and 140 dG as critical developmental time points where the differences are the largest before returning back to similar relative abundances at 165 dG in both groups (Fig. 2). The return of these genes to similar abundances before delivery may also demonstrate the fetus’ ability to adapt to environmental challenges and maintain seemingly normal development. At the same time, however, the return of these genes to similar relative abundances could explain why some of the effects of fetal reprogramming remain latent until later in life and emphasizes the potential importance of maternal diet during the 120 and 140 dG window where these particular transcriptional differences were most pronounced in the fetal liver.

The genes specifically identified as altered between groups fell into two biological networks (Fig. 3). A primary biological network dysregulated between groups was fatty acid β-oxidation (MCODE score = 5.64, nodes = 12, seed = ACSL5, *p* = 7.6 × 10^−11^). While it is difficult to infer the direction of biological effect from RNA-seq data (because of the lag effect between transcription and biological function as well as the potential for some genes to inhibit function or enhance function in different scenarios), it appears that expression of fatty acid β-oxidation genes is almost completely downregulated during 120 and 140 dG by MNR. All of these genes demonstrated decreased relative expression in the MNR group at 120 and 140 dG. This potentially aligns with a switch to a more carbohydrate-mediated energy metabolism and resource storage, as suggested by our earlier findings of increased liver glycogen storage in MNR fetal livers.^3^ This would suggest a transition from a steady-state energy metabolism reliant on a constant supply of fatty acids to a more rapidly adjustable use of gluconeogenesis and glycolysis to maintain the energy needs for the developing fetus, especially the brain. Varying availability of fatty acids from the undernourished mother could be the signal to downregulate the expression of fatty acid β-oxidation related genes in the fetal liver and trigger a switch to carbohydrate utilization and enhanced glycogen storage for future use. After birth, fatty acid β-oxidation plays an essential role in energy metabolism, but for a long time it was thought that only glucose was the primary energy source in the fetus. Recently, fatty acid β-oxidation has been discovered to have an active role during fetal growth – particularly during maternal undernutrition during late gestation.^19–22^ In other animal models, this switch has been demonstrated to lead to severe lipid metabolism disorders and impaired fetal development.^23,24,22^ Fatty acids, in particular, are thought to play a large role in the metabolic reprogramming of the fetus, and the identification of the fatty acid oxidation pathway here coincides with other findings related to maternal NR.^19–21,25,26^

The other main biological network we identified is related to the control of RNA splicing (MCODE score = 4.71, nodes = 15, seed = SRSF2, *p* = 2.7 × 10^−10^). We found that nearly all of these splicing-related genes are down-regulated in the MNR samples at 120 and 140 dG. Perturbation of splicing factors can lead to dramatic differences in splicing, and many of the genes identified in this network are required for carrying out specific splicing activity. Loss of function mutations or knockouts of these genes have been shown to lead to a loss in splicing fidelity which results in an increase in the production of novel (and potentially unwanted or dysfunctional) splice variants created by atypical intron retention or exon skipping.^27–31^ Aberrant splicing caused by mutations in splicing factors often lead to uncontrolled cell growth and eventually tumor adaptability which is why loss of function mutations in splicing factors are commonly associated with cancers.^27–32^ In fact, two of the key genes identified in the networks here - IDH1 and SRSF2 – are recurrently mutated and are actually definitive of certain cancers.^27–29,33,31^ The other key gene identified, PRKAR1A, when mutated, leads to overactive protein kinase A which has been found to phosphorylate splicing factors (among many other roles).^34–36^ Interestingly, PRKAR1A mutations result in Carney complex which is characterized by an increased risk of several types of tumors and significant phenotypic heterogeneity.^34–36^ Similarly, changes in the expression or function of the other splicing-related genes identified in our network are often connected to aberrant splicing and a variety of cancers. These include TRA2B, MBNL1, CCNL2, RAVER1, the HNRNP family, and PRPF4B.^29,37–41^ On a less dramatic scale than loss of function mutations, we propose that the downregulation of these genes caused by decreased nutrient availability in the fetal liver may be the fetus’ way of producing a wider variety of gene isoforms as a potential mechanism of environmental adaptation.

To further test this, we leveraged our RNA-sequencing data to examine if these differentially expressed genes lead to modified differential splicing patterns in other genes in the fetal liver. We observed an overall increase in the number of splice variants for a majority of genes in the MNR samples at 120 and 140 dG (p = 4.65 × 10^−13^) (Fig. 3). This implies splicing was generally increased for most genes in the MNR animals at 120 and 140 dG (Fig. 3; Figs S3–S4). By altering the expression amounts of the splicing-related genes, the MNR fetal livers are increasing the number of splice variants and thereby the potential number of functional protein isoforms. This observation would suggest that individuals, particularly early in development, respond to challenges (such as MNR) by increasing the variety of genes that are expressed, upregulating the production of alternative splice variants and protein isoforms, and expanding their repertoire of proteins to modify and potentially diversify molecular functions. We speculate this may be a cellular response of cells, organs, and organisms to stressors that is intended to find novel ways of dealing long-term with an unfamiliar challenge, similar to strategies reported for yeast in response to the environment.^42^ Similar findings have also been reported in other organisms like shrimp and plants in response to environmental stressors such as nutritional changes and drought.^43–47^ When faced with changes in nutrients during development, including reduced free fatty acids, less glucose, or other more complex metabolic challenges during gestation, the fetal liver cells are lessening control of the highly regulated splicing machinery. In some individuals this strategy may pay off and the system will successfully adapt to the challenges at hand. Increased splicing, particularly if it is permanently incorporated into the way the individual responds to these challenges, could help the individual become more resilient to similar challenges in the future. However, the adaptation that was helpful during development could also lead to unwanted consequences later in life. A potential long-term switch to the preferential expression of a different protein isoform, triggered by increased splicing during this critical fetal developmental window, could have unintended consequences that confer more harm than benefit in adults. Each of these proposed scenarios are unconfirmed hypotheses on how developmental fetal programming may impact long-term health, but may have considerable implications for why some individuals are more resilient to health complications later in life than others and merit further exploration.

The specific splicing factors SRSF2, HNRNPC, and HNRNPH1 have all been previously demonstrated to significantly decrease in abundance with age across multiple tissues, including liver in both humans and mice.^48,49^ This suggests that this observed alteration of splicing may not only be a response to (nutritional) challenges during fetal development, but could also represent a systemic response to physiological challenges related to aging, and a similar attempt of cells, tissues, and organs to explore alternative paths to avoid aging-related complications. This would also suggest that some of these splicing-related changes we observe in fetal livers may be part of biological processes that are related to advanced aging in these MNR offspring. Aging itself is a primary risk factor for most chronic human diseases like type 2 diabetes, hypertension, and arteriosclerosis. This would need much more in-depth consideration, but the concept of programming related changes in aging has become a new and rewarding research field, and the idea of an infant already demonstrating signs of advanced aging at birth (or employing molecular mechanisms to counteract aging-related complications this early in life) because of challenges faced in utero is proving a unique concept to explore.

Overall, we have discovered numerous transcripts that are differentially expressed during the development in the MNR and control primate fetal liver. These transcripts are primarily involved in two main pathways – fatty acid β-oxidation and regulation of RNA splicing – which demonstrate the most drastic differences at 120 dG and 140 dG. This suggests that between 120 and 140 dG there is critical developmental window where alterations to fatty acid β-oxidation and RNA splicing are dependent on the intrauterine environment. The changes to these pathways seem to disappear immediately pre-term, but may have latent effects later in life. Lastly, we also demonstrated that the differences in the abundance of the RNA splicing control genes are reflected in the increased numbers of RNA splice variants present in the MNR fetal livers compared to the controls. These findings may have considerable implications for how the fetus attempts to circumvent challenges posed to it by maternal undernutrition. More broadly, they raise novel questions about the biological mechanisms underlying resilience and responses to challenges, be it acute nutritional challenges during fetal development or common aging-related later-life challenges. Further investigation of splicing mechanisms as a means of generating molecular diversity and dealing with adversity are required, but if found to exist, such mechanisms may shift the way we understand fetal development, aging, susceptibility, and resilience to disease.

## Supplementary Material

1
