# Supplementary material for: Moderate maternal nutrient reduction in pregnancy alters fatty acid oxidation and RNA splicing in the nonhuman primate fetal liver
Source: J Dev Orig Health Dis. Author manuscript; Available in PMC 2023 Dec 1. (PMC10202844; doi:10.1017/S204017442300003X)
Supplement: 1 [file NIHMS1873073-supplement-1.docx]

**Supplemental Information**

**
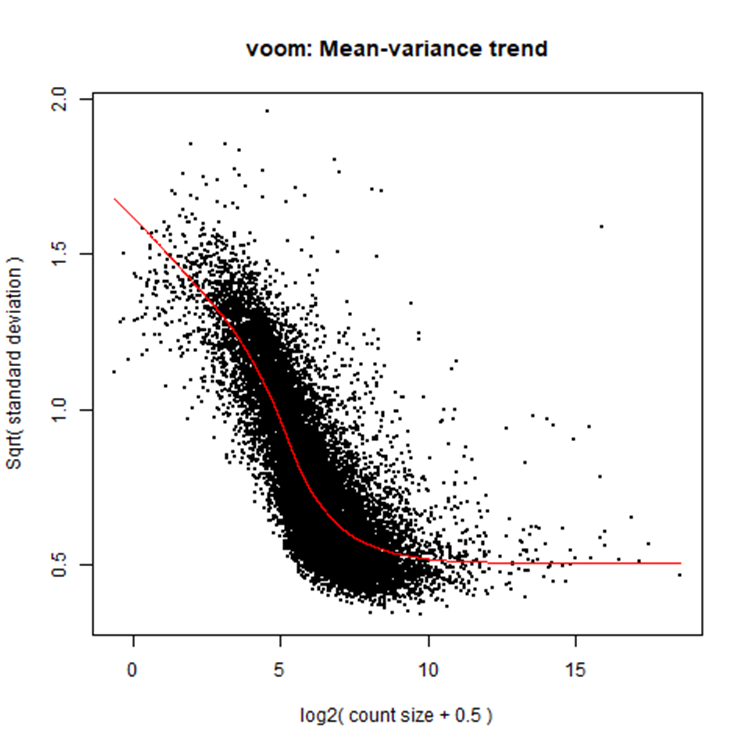
**

**Figure S1 – Mean-variance trend fit.** The relationship between average expression and the estimated variant for each gene after filtering. The smoothed curve is used to obtain weights for each gene and sample that used in the final linear model.

**
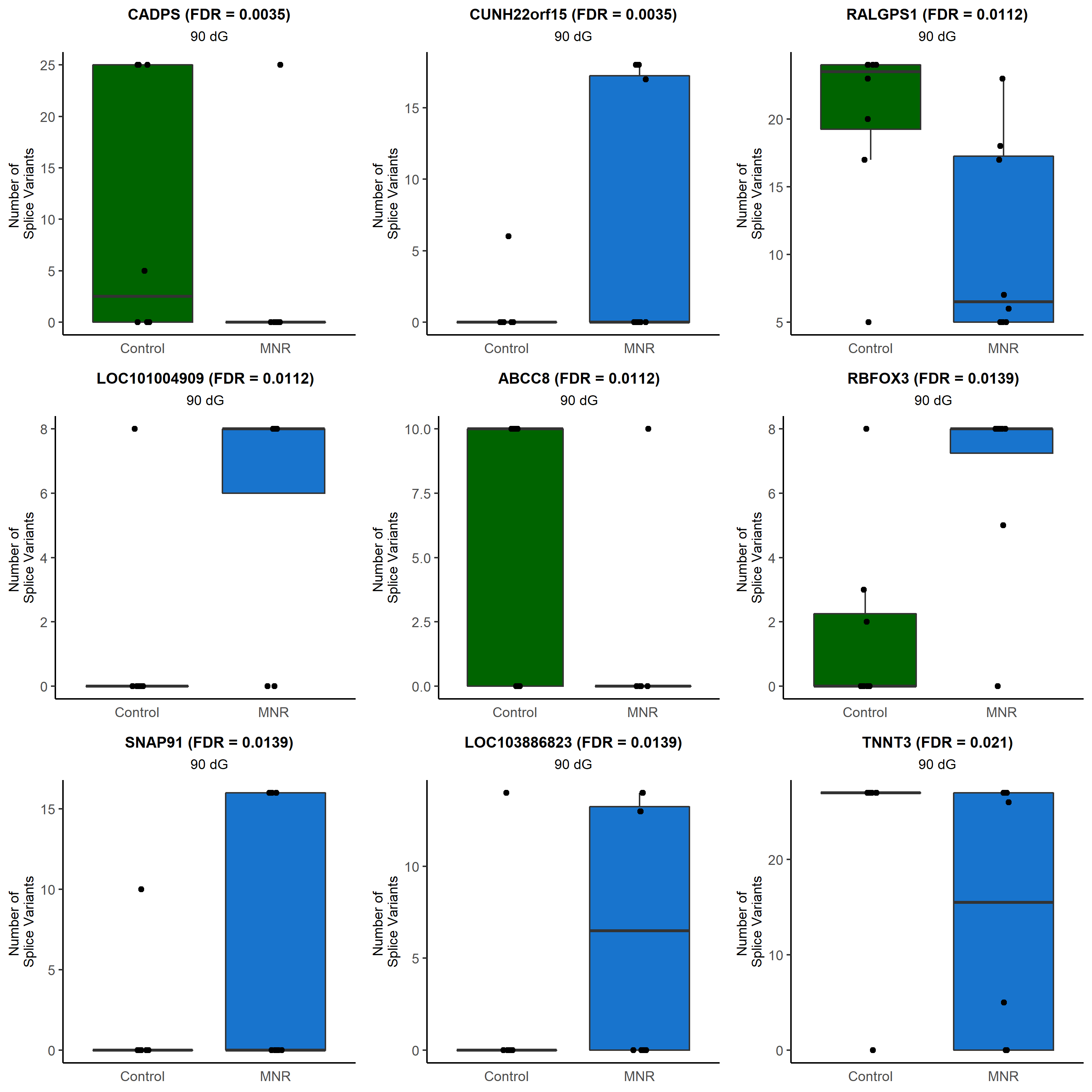
**

**Figure S2 – Splicing differences at 90dG.** Standard boxplots of the number of splice variants for each gene that demonstrated significant differences between MNR and control animals at 90dG. In order to be counted, a splice variant had to have at least one read present for that particular gene’s splice variant. The center line represents the median. The lower and upper box limits represent the 25% and 75% quantiles, respectively. The whiskers extend to the largest observation within the box limit ±1.5 × interquartile range. FDR p-values are reported in parentheses next to the gene names.

**
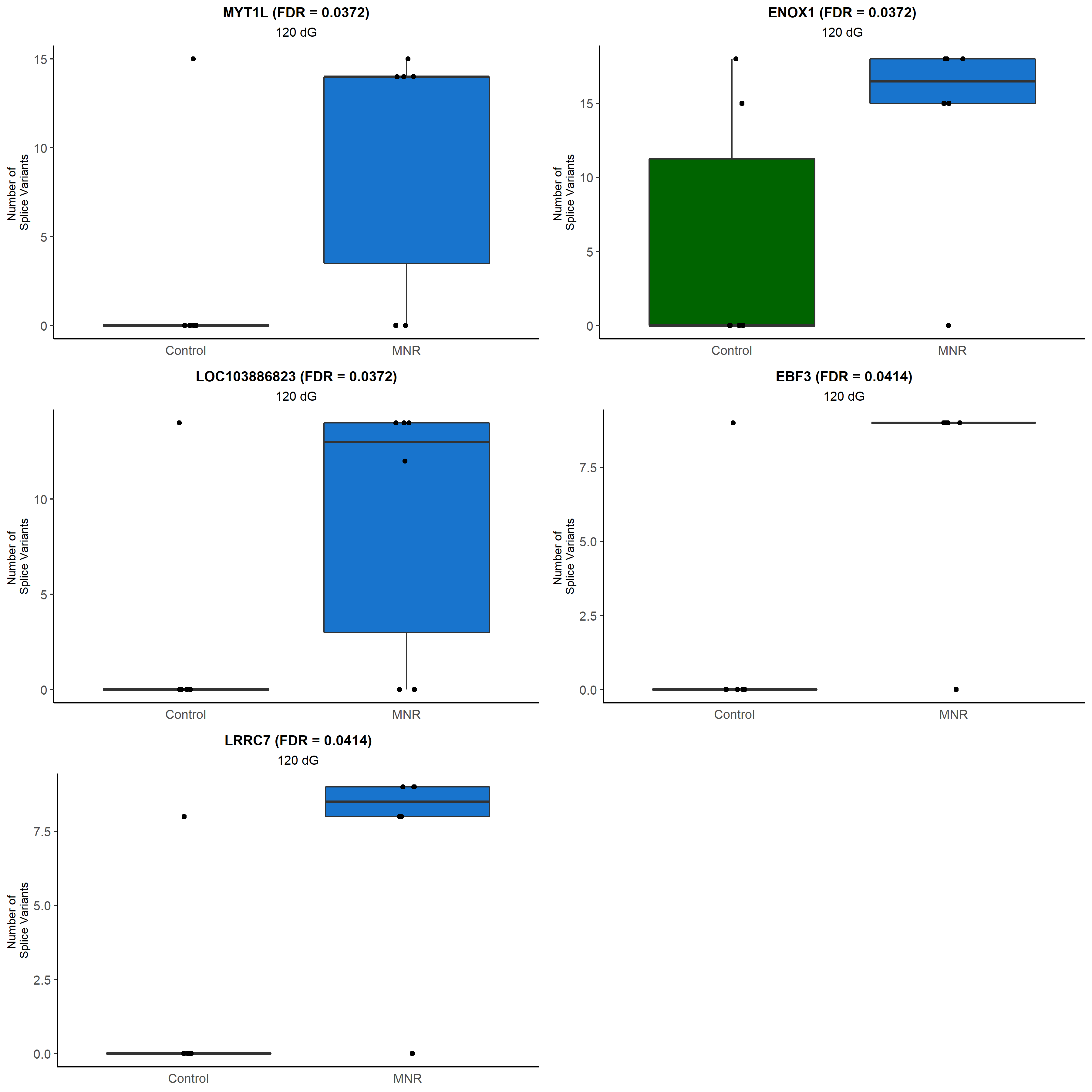
**

**Figure S3 – Splicing differences at 120dG.** Standard boxplots of the number of splice variants for each gene that demonstrated significant differences between MNR and control animals at 120dG. In order to be counted, a splice variant had to have at least one read present for that particular gene’s splice variant. The center line represents the median. The lower and upper box limits represent the 25% and 75% quantiles, respectively. The whiskers extend to the largest observation within the box limit ±1.5 × interquartile range. FDR p-values are reported in parentheses next to the gene names.

**
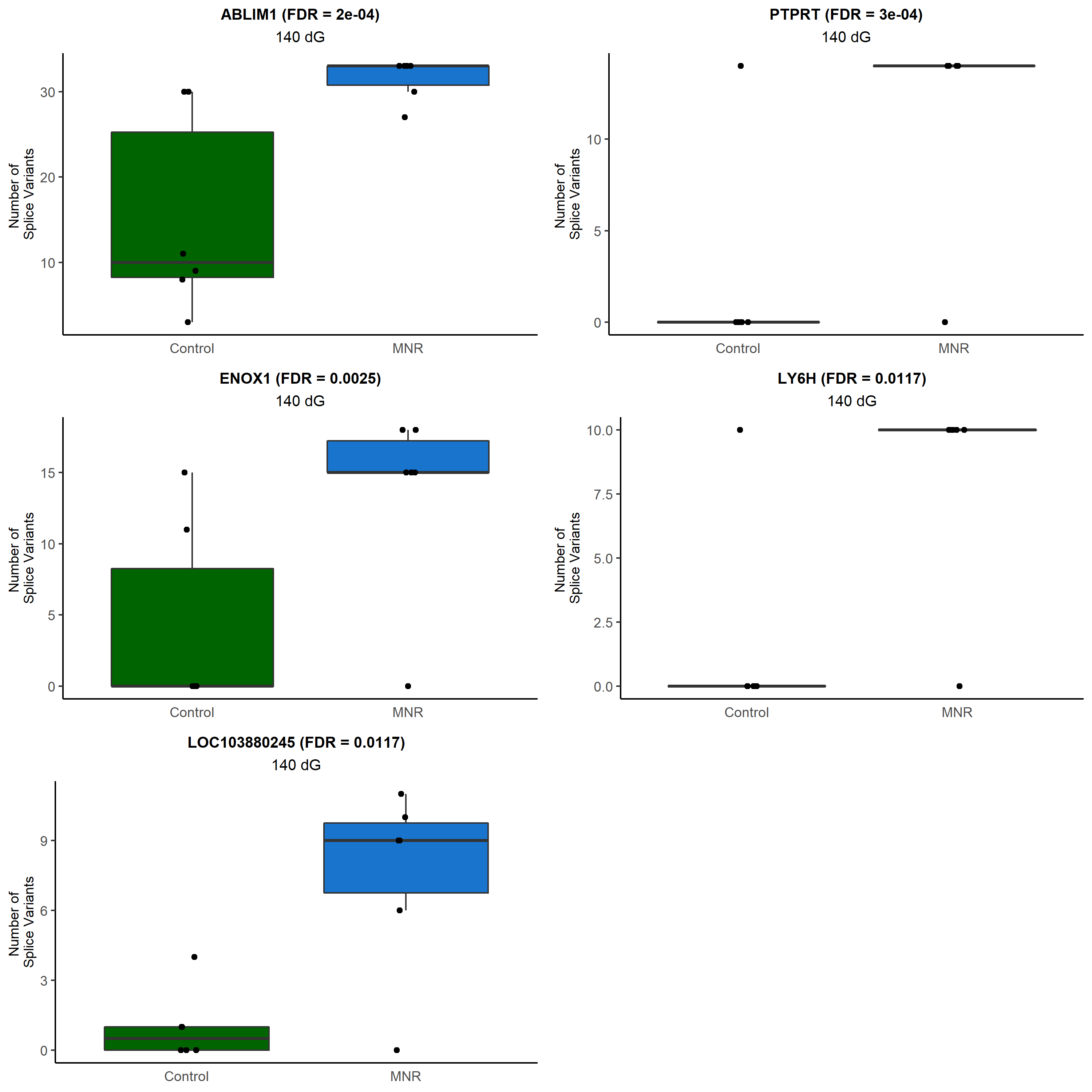
**

**Figure S4 – Splicing differences at 140dG.** Standard boxplots of the number of splice variants for each gene that demonstrated significant differences between MNR and control animals at 140dG. In order to be counted, a splice variant had to have at least one read present for that particular gene’s splice variant. The center line represents the median. The lower and upper box limits represent the 25% and 75% quantiles, respectively. The whiskers extend to the largest observation within the box limit ±1.5 × interquartile range. FDR p-values are reported in parentheses next to the gene names.

**
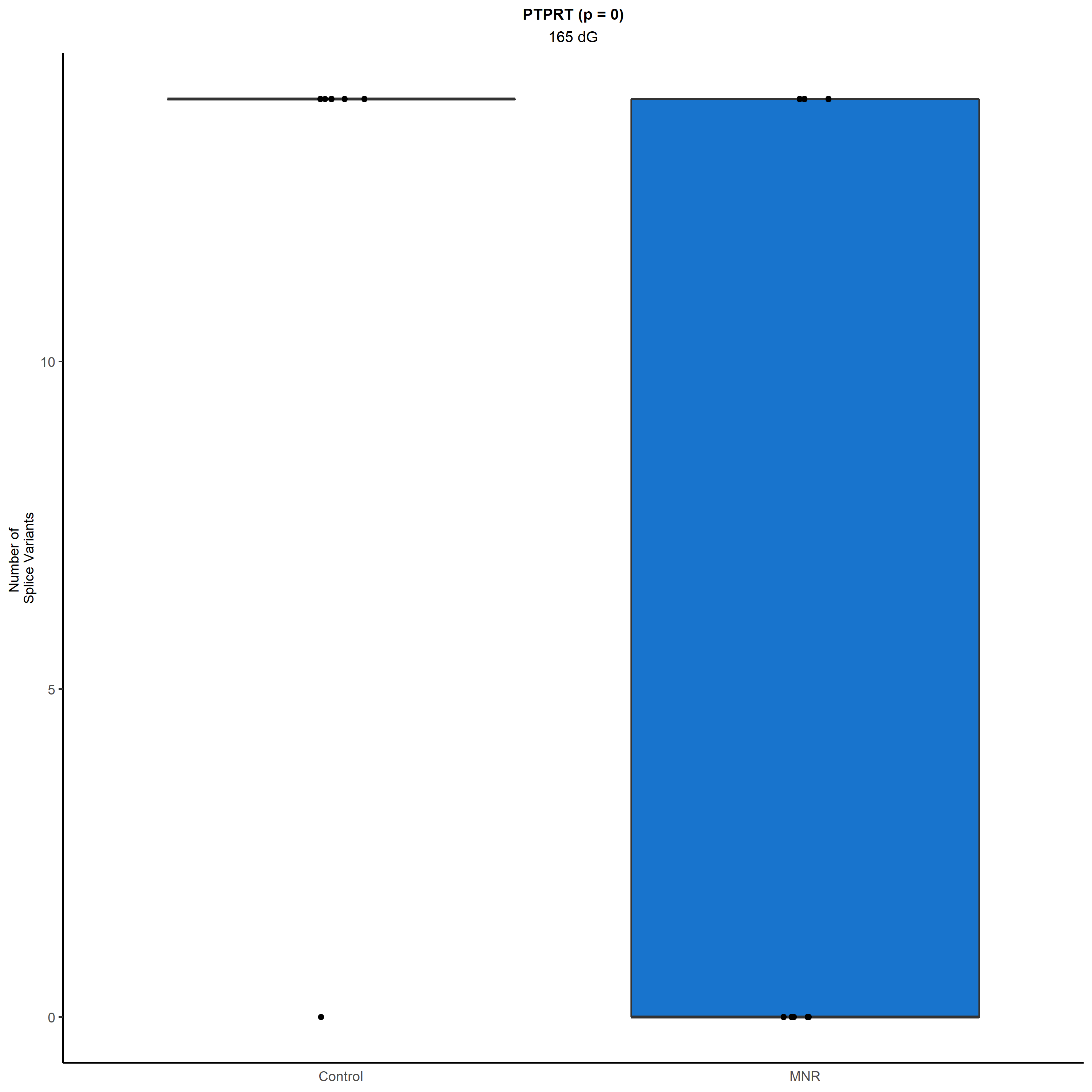
**

**Figure S4 – Splicing differences at 165dG.** Standard boxplots of the number of splice variants for each gene that demonstrated significant differences between MNR and control animals at 165dG. In order to be counted, a splice variant had to have at least one read present for that particular gene’s splice variant. The center line represents the median. The lower and upper box limits represent the 25% and 75% quantiles, respectively. The whiskers extend to the largest observation within the box limit ±1.5 × interquartile range. FDR p-values are reported in parentheses next to the gene names.

**Table S1 – Differentially expressed transcripts (FDR < 0.1) in MNR animals.** Transcripts are listed along with gene symbol, effect size, p-value, FDR adjusted p-value, and from which contrast they were significant. Repeated genes across both contrasts are in bold to highlight how much overlap exists between the two contrasts.

| Transcript | Gene Symbol | Effect Size [95% CI] | p-value | FDR | Contrast |
| --- | --- | --- | --- | --- | --- |
| XM_009201803.1 | **LOC103882414** | -1.31 [-1.77:-0.86] | 3.50x10^-07^ | 0.005 | MNR |
| XM_009193929.3 | **SLC25A42** | -0.86 [-1.24:-0.48] | 2.92x10^-05^ | 0.085 | MNR |
| XM_003911357.4 | HSDL2 | -0.63 [-0.91:-0.35] | 2.99x10^-05^ | 0.085 | MNR |
| XM_003911822.3 | **NMRK1** | -0.8 [-1.15:-0.45] | 3.03x10^-05^ | 0.085 | MNR |
| XM_003908369.3 | **HADHB** | -0.72 [-1.05:-0.4] | 3.55x10^-05^ | 0.085 | MNR |
| XM_021934972.1 | **ST6GAL1** | -1.56 [-2.27:-0.85] | 4.96x10^-05^ | 0.085 | MNR |
| XM_017953498.2 | CDH1 | -0.66 [-0.97:-0.36] | 5.85x10^-05^ | 0.085 | MNR |
| XM_021938982.1 | **ACSL1** | -1 [-1.46:-0.54] | 6.20x10^-05^ | 0.085 | MNR |
| XM_003912220.3 | **ACADVL** | -0.58 [-0.85:-0.31] | 7.27x10^-05^ | 0.085 | MNR |
| XM_009188596.3 | **GALT** | -0.77 [-1.13:-0.41] | 7.45x10^-05^ | 0.085 | MNR |
| XM_009209923.1 | IVD | 0.57 [0.3:0.84] | 7.97x10^-05^ | 0.085 | MNR |
| XM_021923228.1 | **ERGIC2** | -0.6 [-0.89:-0.32] | 8.52x10^-05^ | 0.085 | MNR |
| XR_001905774.2 | DPYS | 0.0013 [0.0008:0.0018] | 5.32x10^-06^ | 0.044 | Quadratic |
| XM_009201803.1 | **LOC103882414** | 0.001 [0.0006:0.0014] | 7.25x10^-06^ | 0.044 | Quadratic |
| XM_003908369.3 | **HADHB** | 0.0007 [0.0004:0.001] | 1.47x10^-05^ | 0.044 | Quadratic |
| XM_009201909.3 | **ST6GAL1** | 0.0014 [0.0008:0.0019] | 1.54x10^-05^ | 0.044 | Quadratic |
| XM_003913970.3 | EDNRB | 0.0018 [0.001:0.0025] | 1.89x10^-05^ | 0.044 | Quadratic |
| XM_021934975.1 | BCL6 | 0.0008 [0.0004:0.0011] | 3.19x10^-05^ | 0.044 | Quadratic |
| XM_017961245.2 | LOC101014452 | 0.0013 [0.0007:0.0019] | 3.22x10^-05^ | 0.044 | Quadratic |
| XM_003898170.3 | VNN1 | 0.0008 [0.0005:0.0012] | 3.29x10^-05^ | 0.044 | Quadratic |
| XM_021938982.1 | **ACSL1** | 0.001 [0.0005:0.0014] | 3.59x10^-05^ | 0.044 | Quadratic |
| XM_021937511.1 | PHIP | 0.0009 [0.0005:0.0013] | 4.92x10^-05^ | 0.045 | Quadratic |
| XM_003907888.3 | IDH1 | 0.001 [0.0006:0.0015] | 5.14x10^-05^ | 0.045 | Quadratic |
| XM_003912220.3 | **ACADVL** | 0.0006 [0.0003:0.0008] | 5.46x10^-05^ | 0.045 | Quadratic |
| XM_009193929.3 | **SLC25A42** | 0.0007 [0.0004:0.0011] | 6.14x10^-05^ | 0.045 | Quadratic |
| XM_009205936.2 | SHPRH | 0.0006 [0.0003:0.0009] | 6.23x10^-05^ | 0.045 | Quadratic |
| XM_021929339.1 | PRKAR1A | 0.0019 [0.001:0.0027] | 6.28x10^-05^ | 0.045 | Quadratic |
| XM_021934131.1 | HACD2 | 0.001 [0.0005:0.0014] | 6.99x10^-05^ | 0.046 | Quadratic |
| XM_017954419.2 | F9 | 0.001 [0.0005:0.0015] | 7.10x10^-05^ | 0.046 | Quadratic |
| XM_021923228.1 | **ERGIC2** | 0.0006 [0.0003:0.0008] | 7.74x10^-05^ | 0.048 | Quadratic |
| XM_009209825.3 | MTMR10 | 0.0008 [0.0004:0.0012] | 8.17x10^-05^ | 0.049 | Quadratic |
| XM_003913834.3 | SLC25A30 | 0.0006 [0.0003:0.0008] | 1.19x10^-04^ | 0.065 | Quadratic |
| XM_009211314.2 | CIDEB | 0.0004 [0.0002:0.0006] | 1.22x10^-04^ | 0.065 | Quadratic |
| XM_003892503.4 | SEC22B | 0.0011 [0.0005:0.0016] | 1.48x10^-04^ | 0.076 | Quadratic |
| XM_009188596.3 | **GALT** | 0.0007 [0.0003:0.001] | 1.56x10^-04^ | 0.078 | Quadratic |
| XM_021924041.1 | WDFY1 | 0.0009 [0.0004:0.0013] | 1.80x10^-04^ | 0.087 | Quadratic |
| XM_003911822.3 | **NMRK1** | 0.0007 [0.0003:0.001] | 1.93x10^-04^ | 0.090 | Quadratic |
| XM_009208387.3 | NNT | 0.0013 [0.0006:0.002] | 2.28x10^-04^ | 0.095 | Quadratic |
| XM_003917166.3 | COG4 | -0.0007 [-0.001:-0.0003] | 2.32x10^-04^ | 0.095 | Quadratic |
| XR_001905819.2 | COLEC10 | 0.0013 [0.0007:0.002] | 2.34x10^-04^ | 0.095 | Quadratic |
